# Effects of drought stress induced by D-Mannitol on the germination and early seedling growth traits, physiological parameters and phytochemicals content of Tunisian squash (*Cucurbita maxima*Duch.) landraces

**DOI:** 10.3389/fpls.2023.1215394

**Published:** 2023-07-31

**Authors:** Wassim Saadaoui, Neji Tarchoun, Insaf Msetra, Ourania Pavli, Hanen Falleh, Chadha Ayed, Roua Amami, Riadh Ksouri, Spyridon A. Petropoulos

**Affiliations:** ^1^ Research Laboratory LR21AGR05, High Agronomic Institute of ChottMariem, University of Sousse, Sousse, Tunisia; ^2^ Laboratory of Genetics and Plant Breeding, Department of Agriculture, Crop Production and Rural Environment, University of Thessaly, Volos, Greece; ^3^ Laboratory of Aromatic and Medicinal Plant, Centre of Biotechnology of Borj Cedria, Tunis, Tunisia; ^4^ Laboratory of Vegetable Production, Department of Agriculture, Crop Production and Rural Environment, University of Thessaly, Volos, Greece

**Keywords:** water stress, *Cucurbita maxima* Duchesne, seed germination, seedling vigor, total phenols content, chlorophyll fluorescence, carotenoids

## Abstract

**Introduction:**

Drought stress is one of the most devastating environmental stressors, especially in the arid and semi-arid regions of the world. Considering the major constraints that drought stress poses to crop production and the consequent yield losses in food crops, breeding for climate-resilient crops is an efficient means to mitigate stress conditions.

**Materials and methods:**

This study aimed at evaluating the response of four squash (*Cucurbita maxima* Duchesne) landraces to drought stress at germination and at plant stage. Drought stress was induced by different concentrations of D-mannitol (-0.24, -0.47 and -0.73 MPa). The tested parameters at germination stage included germination percentage, seedling vigor index, seed water absorbance and seedling growth potential. At the plant stage, leaf chlorophyll and carotenoids content, chlorophyll fluorescence, evapotranspiration, photosynthesis activity and several biomarkers, namely malondialdehyde, proline, total phenols content, total flavonoids content and DPPH radical scavenging activity were evaluated in both roots and leaves.

**Results and discussion:**

Our results indicate a magnitude of drought stress effects reflected via repression of germination and seedling growth as well as adjustments in physiological functions at later growth stages, in a genotype depended manner. Among landraces, "751" and "746" showed better performance, as evidenced by higher seed germination and seedling growth potential even at high stress levels (-0.47 and - 0.73 MPa), whereas "747" was the most sensitive landrace to drought stress at both tested stages. In conclusion, our findings highlight the importance of squash landraces selection for the identification of elite genotypes with increased tolerance to drought stress.

## 1 Introduction

Over the last decades, climate change substantially intensifies drought incidences as a result of warming and the increased evaporation. Drought is undoubtedly one of the most important environmental stressors that severely affects a wide range of major food crops, leading to considerable yield losses, especially in the arid and semi-arid regions. Considering these adverse effects of drought to both plant growth and productivity, breeding for drought tolerant varieties is generally considered as an effective and sustainable means of ensuring economic viability of crops under water deficit conditions. The mitigation of yield losses under drought conditions primarily relies on improving traits that enhance drought tolerance, such as water consumption and water use efficiency (WUE) (Hatfield and Dold, 2019). WUE refers to plant’s ability to cope with moderate or severe soil water deficit and is considered as an important indicator of plant survival under limited water availability (Schulz et al., 2021). The response of plants to water deficit conditions includes a series of morphological, physiological, and biochemical modifications, including changes in photosynthesis, respiration, transpiration processes as well as the accumulation of reactive oxygen species (ROS) (Chevilly et al., 2021; Giordano et al., 2021; Nalina et al., 2021; Zhou et al., 2021). The molecular responses to drought include the activation of antioxidant enzymes and osmolyte accumulation which serve towards the stabilization of membranes, enzymes and proteins, as well as to the protection against oxidative damage through scavenging of ROS (Hasanuzzaman et al., 2020; Begum et al., 2022). Regarding the plant organs level, leaf morphology and functions may promote water use efficiency, thus contributing to mitigation of stress effects, while root characteristics such as length, weight, volume, and density, play a pivotal role in drought tolerance (Kapoor et al., 2020).

In the search to identify eligible strategies to improve drought tolerance of crops, several studies highlight the complexity of plant responses to drought, involving numerous factors such as the growth stage, the duration and severity of stress (Golldack et al., 2014), as well as the polygenic nature of quantitative tolerance traits (Mahmood et al., 2020). Considering that most plant species are drought-sensitive at all stages of their lifecycle, seed germination is considered as the most critical stage since water deficit impairs germination by limiting water imbibition and further reduces seedling vigor, via repressing root emergence and shoot elongation, thus preventing the establishment of a uniform stand (Queiroz et al., 2019; Rosero et al., 2020). As such, the most common plant response to reduced osmotic potential refers to delayed and reduced germination rates(Saberali and Shirmohamadi-Aliakbarkhani, 2020). Such adverse effects have been reported for a plethora of crop species, including maize (Queiroz et al., 2019), sorghum (Cokkizgin et al., 2019), faba bean (Li et al., 2018), cucumber(Mombeini et al., 2021), and melon (Saroj and Choudhary, 2020). Therefore, it is suggested that seed germination and seedling growth potential under stress conditions may enable the early selection of tolerant genotypes, thus enhancing the efficiency of selection procedures for suitable germplasm material to be cultivated or exploited in relative breeding programs (Tarchoun et al., 2022).

In the context of improving complex traits, such as drought tolerance, the research interest is progressively focused on the search of functional markers to be employed in breeding procedures aimed at early selecting for tolerant genotypes. Well known examples of stress indicators are certain amino acids of the group of quaternary amines with osmoprotective functions under water stress conditions, such as proline and glycine betaine(Bankaji et al., 2019). In the same line, plant protection against oxidative damage involves the induction of the activity of antioxidant enzymes(Gill and Tuteja, 2010). Carotenoids serve in multiple functions in plants, including photosynthesis and regulation of redox status(Gill and Tuteja, 2010), while phenolic compounds contribute to plant defense mechanisms against abiotic stress factors (Cheynier et al., 2013).

Given that photosynthesis is one of the most critical metabolic processes, its repression under drought stress adversely affects plant growth and development (Al Hassan et al., 2015). Upon severe drought stress, both in terms of duration and intensity, inhibition of photosynthesis is manifested by changes in chlorophyll content, mainly due to ROS-induced damage of chloroplasts, as well as damages in the whole photosynthetic apparatus (Porcar-Castell et al., 2014). Accordingly, ROS production is mainly driven by excess energy absorption in the photosynthetic apparatus, that might be reversed by degrading the absorbing pigments. Therefore, chlorophyll fluorescence is considered a fast, accurate and non-invasive tool to monitor photosystem II (PSII) (Baker, 2008), while photosynthetic activity can also be assessed since chlorophyll fluorescence can describe and investigate the photosynthetic light processes and quantum conversion by chlorophyll and accessory pigments of chlorophyll-protein complexes (Porcar-Castell et al., 2014).

Among the cultivated *Cucurbita* species, e.g. *C. argyrosperma,C. ficifolia, C. maxima*, *C. moschata* and *C. pepo*, the latter three are the most important in terms of worldwide production. In Tunisia, the squash (*C. maxima*Duch.) germplasm used for commercial cultivation is essentially derived from local landraces, produced by open pollination or farmer mass selection. Given the importance of local squash production, an increasing number of studies focuses on collection, *ex-situ* and *in-situ* conservation, characterization and maintenanceof local germplasm accessions (Hamdi et al., 2017; Hamdi et al., 2019; Tarchoun et al., 2022). Local landraces are often characterized by particularly broad leaves, thus leading to high evapotranspiration and high crop water requirements, since cultivation usually takes place during the warmer months of the year (Hamdi et al., 2017).

In this context, breeding efforts are focused on the selection of drought tolerant landraces so as to ensure economic viability of squash cropping in arid and semi-arid regions, such as Tunisia. Although several studies aimed at understanding the drought stress response in Cucurbita species, mainly *C. pepo* and *C. moschata*, so far (Biareh et al., 2022), there is a gap in relevant research for *C. maxima*. Therefore, this study aimed at (i) investigating the response of four squash local landraces of Tunisia to drought stress at germination stage, (ii) examining the possibilities of early selecting drought tolerant genotypes and (iii) determining the effects of drought stress at later plant growth stages. For this purpose, drought stress was imposed by different water potential (0, -0.24, -0.47 and -0.73 MPa). During germination, the evaluation of drought tolerance was based on traits related to germination and seedling growth (germination percentage, seedling vigor index, root and shoot length, root and shoot fresh weight, root volume and seed water absorbance). At later growth stages, the response of plants to stress was assessed using physiological (chlorophyll fluorescence, chlorophylls and carotenoid content, evapotranspiration, photosynthesis activity) and biochemical parameters (malondialdehyde (MDA), free proline, total phenolic compounds and total flavonoid content) and DPPH radical scavenging activity of roots and leaves.

## 2 Materials and methods

### 2.1 Plant material

Four landraces were selected for further evaluation of their response to D-mannitol induced drought stress, based on relevant assessment data related to the response of fifteen squash landraces to varying salt stress levels (Tarchoun et al., 2022). The genotypes under study consisted of four local landraces representing the main types of cultivated squash, namely Batati orange (“746”), Galaoui (“747”), Karkoubi Orange (“748”) and Bejaoui Green (“751”) (**Table 1**). Each landrace was assigned with passport data and an inventory number, according to the National Gene Bank of Tunisia, while full details are available at the Germplasm Resources Information Network - GRIN (http://www.tn-grin.nat.tn/gringlobal/search.%20aspx, accessed on 15 February 2022). The description of fruit morphology was performed based on the European Cooperative Program for Plant Genetic Resources (ECPGR) list of descriptors for *Cucurbita* spp. (ECPGR, 2008).

**Table 1:** Description of the Tunisian squash landraces employed in this study.

Landrace Inventory	Local Name	Origin	Short Description
NGBTUN746 (“746”)	Batati Orange	Siliana (Sidi Hamada)	Globular fruit, orange skin, light orange flesh
NGBTUN747 (“747”)	Galaoui	Ariana (KalaaAndalous)	Raised fruit with basal tip, green skin, green flesh
NGBTUN748 (“748”)	Karkoubi Orange	Sousse (SidiBouali)	Flattened fruit, dark yellow skin, yellow flesh
NGBTUN751 (“751”)	Bejaoui Green	Siliana (Sidi Hamada)	Flattened fruit, dark green skin, light green flesh

### 2.2 Drought stress treatments at germination stage

The present research was conducted at the Department of Horticulture, Vegetable Laboratory, High Agronomic Institute of Chott Mariam, Tunisia. Drought stress was induced by D-mannitol at different stress levels (0, -0.24, -0.47 and -0.73 MPa), while the response of the studied *C. maxima* genotypes was assessed based on traits related to germination and seedling growth (Tarchoun et al., 2022). The osmotic potential of the tested treatments was as follows: 0 MPa; - 0.24 MPa; - 0.47 MPa and - 0.73 MPa. One hundred and sixty (160) seeds per landrace were collected from mother plants protected from cross-pollination based on size homogeneity. Then, they were subsequently surface-sterilized, using 1% hypochlorite/H_2_O solution under gentle agitation for 5 min, and washed 4 times with excess of sterile water. Sterilized seeds were initially primed in gibberellic acid solution (1.5 mM GA_3_) for 24h, to stimulate germination, and then rinsed in sterile water. Ten seeds of each accession were placed in a Petri dish (90 mm) and lined with two layers of filter paper soaked with the appropriate D-mannitol concentration, while four Petri dishes per treatment were used. Seeds were allowed to germinate under controlled conditions (28 ± 2°C, 16 h light/8 h dark, 50 ± 5% relative humidity). Seeds were regularly monitored and 3 mL of the respective D-mannitol solution were added daily to ensure continuous germination.

### 2.3 Parameters for evaluation of drought tolerance at germination stage

Drought tolerance was evaluated on the basis of various parameters related to seed germination and seedling growth potential under the tested drought stress levels (Tarchoun et al., 2022). Specifically, genotype evaluation for drought tolerance was performed on the basis of germination percentage (GP), seed water absorbance (WU), seedling vigor index (SVI), root and shoot length (RL, SL), root and shoot fresh weight (RFW, SFW) and ratio of shoot length to root length (SL/RL).

Germination percentage was scored daily for a period of 15 days, until no more germinated seeds were recorded. Seeds were considered as germinated when the radicle had emerged from the seed coat and had a length of at least 2 mm. Seed germination percentage was determined according to the formula:


$$Germination\;percentage\;(GP)=\frac{number\;germinated\;seeds}{number\;of\;total\;seeds}\times 100$$

(Scott et al., 1984).

WU (%) and SVI (%) were estimated at the 15^th^ day, according to the formula:


$$Water\;seed\;absorbance\;(WU)=\frac{initial\;seed\;weight}{seed\;weight\;following\;water\;absorbance}\times 100$$

(Partheeban et al., 2017),

Seedling vigor index (SVI) = (Root length + Shoot length) × GP % (Ashraf et al., 2021).

SL (cm), RL (cm), SL/RL ratio, as well as RFW (g) and SFW (g) were estimated at the 15^th^ day after treatment initiation in ten seedlings per treatment and accession (Tarchoun et al., 2022). The fresh weight (FW) of shoots and roots was recorded at harvest, and shoot and root dry weight (DW) was measured after oven drying (Memmert, Buchenbach, Germanny) at 70°C for 48 h. For the determination of the root volume (RV) of untreated and stressed plants, a beaker was filled with dH_2_Oin a constant volume (V_1_) and the final volume (V_2_) was recorded after placing the root in the beaker. Root volume (RV) was determined using the formula:


$$\text{RV }(\text{c}{\text{m}}^{3})={\text{V}}_{2}-{\text{V}}_{1}$$

### 2.4 Drought stress treatments at the plant stage

Fifteen days after the start of the experiment, 15 selected seedlings for each landrace and stress level (60 seedlings per accession) were planted into 2 L pots containing peat and soil to ensure good drainage and prevent water logging. Planted seedlings were transferred to the greenhouse under controlled conditions and watered regularly at 2-day intervals for approximately 7 weeks, with solutions differing in D-mannitol concentration (0, -0.24, -0.47 and -0.73 MPa). Once a week, plants were also fertilized with nutrient solution (20-20-20; N-P-K; 2g/L/plant). After 45 days, the plant response to drought stress was assessed on the basis of physiological parameters, e.g. chlorophyll fluorescence, content of chlorophylls a, b and carotenoids, evapotranspiration (RET) and photosynthetic activity (PA), and biochemical parameters, e.g. malondialdhehyde (MDA), free proline, total phenols and total flavonoids content and DPPH radical scavenging activity, the latter determined in both roots and shoots.

#### 2.4.1 Chlorophyll fluorescence, PhotosyntheticActivity and Real Evapotranspiration

Chlorophyll fluorescence was measured on fully expanded healthy leaves using a fluorometer (Plant Stress Kit, Opti-Sciences model, NH, USA). The dark adaptation period and the level of saturating light were determined before measurements. After dark adaptation of leaves for 15 min, the maximum fluorescence yield (F_m_), evaluated at 6000 µmol saturation pulse, and minimum leaf fluorescence yield (F_0_) of the leaves were first determined. The values of minimum (F_0_) and maximum (F_m_) fluorescence,as well as those for the F_v/_F_m_ quantum ratio were also determined using Fluorometer (Plant kit stress, Opti-Sciences model, NH, USA).Photosynthetic activity (PA) (µmol photons m^-2^ s^-1^) and real evapotranspiration (RET) (mm H2O/day)were measured on the fourth fully formed leaf from the apex in a clear day using Fluorometer (Plant kit stress, Opti-Sciences model, NH, USA).

#### 2.4.2 Chlorophyll and carotenoids content

Chlorophyll content was determined according to the method of (Porcar-Castell et al., 2014). Briefly, 0.1 g of leaf tissue were ground with 10 mL of 80% acetone. Following filtration, the solutions were incubated in the dark to avoid photo-oxidation. The chlorophyll content (Chlorophyll a (Chla) and Chlorophyll b (Chlb)) of the filtered solution was measured using a UV-visible spectrophotometer (Evolution 210, Thermo Scientific, Abingdon, UK) at 645 and 663 nm, respectively, whereas carotenoids content was determined at 470 nm (Curtis and Shetty, 1996). Calibration of the apparatus was performed using 80% acetone. The relationship between concentration (mg/g fresh matter) and optical density was determined according to the following formulas (Arnon, 1949):


$$\text{Chla }(\text{mg}/\text{g})=12.7\times {\text{D}}_{663}-2.59\times {\text{D}}_{645}$$

$$\text{Chlb }(\text{mg}/\text{g})=22.9\times {\text{D}}_{645}-4.68\times {\text{D}}_{663}$$

, $$\text{Ccar }(\text{mg}/\text{g})=[(5\times {\text{D}}_{470})\times (3.19\times \text{Chla}))+(130.3\times \text{Chlb})]/200,$$

where D refers to absorbance at the corresponding nanometers.

#### 2.4.3 Free proline content

Free proline content was determined using the method of (Monneveaux and Nemmar, 1986). Briefly, 100 mg of fresh leaf tissue were placed in 5 mL of 40% methanol and the mixture was heated for 1 h in a water bath at 85°C. After cooling, 1 mL of the extraction solution was added to 1 mL of acetic acid, 25 mg of ninhydrin and 1 mL of the mixture [dH_2_O+ acetic acid + orthophosphoric acid of density 1.7 (120:300:80, v/v/v)]. Following incubation for 30 min in a water bath (100°C) and subsequent cooling, 5 mL of toluene were added to the samples. The upper phase containing proline was recovered and its optical density was determined at 528 nm using a UV-Vis spectrophotometer (Evolution 210, Thermo Scientific, Abingdon, UK). Proline concentration was calculated using a standard curve created using stock solutions ranging from 0 to 2.5 mg/mL of L-proline. Proline contents were expressed as µg/mg FW.

#### 2.4.4 Malondialdehyde

Two hundred mg of fresh leaves were ground and homogenized in 1 mL of 0.1% trichloroacetic acid (TCA) (Hnilickova et al., 2021). Following centrifugation at 15000 rpm for 10 min at 4°C, 0.5 mL of the supernatant was mixed with 1 mL of 0.5% thiobarbituric acid (TBA). The solution was heated to 95°C for 30 min, cooled and centrifuged at 500 rpm for 30 min and the absorbance was measured at 532 nm using a UV-Vis spectrophotometer (Evolution 210, Thermo Scientific, Abingdon, UK). The calibration curve was prepared using TBA (0.5%) and TCA (20%). MDA content was expressed as µmol g^-1^ FW.

#### 2.4.5 Total phenolic compounds content

Total phenolic content was assayed using Folin-Ciocalteu colorimretic method according to (Blainsk et al., 2013). Briefly, 0.125 ml of methanolic extract solution was mixed with 0.5 ml of dH_2_O and 0.125 ml of the Folin-Ciocalteu reagent. Following incubation for 1 min, 1.25 mL of7% sodium carbonate (Na_2_CO_3_) solution were added. After incubation for 120 min in the dark, the absorbance was measured at 760 nm using a UV-Vis spectrophotometer (Evolution 210, Thermo Scientific, Abingdon, UK). The TPC was expressed as mg gallic acid equivalent (GA) per 100 mg dry weight (DW).

#### 2.4.6 Total flavonoid content

Total flavonoids content of the methanolic extract solution was determined using the aluminum chloride assay (Zhishen et al., 1999). A total of 0.5 mL methanolic extract was mixed with 2.5 mL of dH_2_O and 0.15 mL of 5% sodium nitrite (NaNO_2_) solution. The mixture was incubated for 5 min before adding 0.3 ml of 10% aluminum chloride (AlCl_3_) and 1 mL of 1 M sodium hydroxide (NaOH) solution. Following incubation for 15 min, the absorbance was measured at 510 nm using a UV-Vis spectrophotometer (Evolution 210, Thermo Scientific, Abingdon, UK). The TFC was expressed as mg quercetin equivalent (QE) per 100 mg dry weight (DW).

#### 2.4.7 DPPH radical scavenging activity

Antioxidant activity was evaluated as the scavenging activity of 1,1-diphenyl-2-picrylhydrazyl (DPPH) radical (Ferreira et al., 2007). A total of 0.5 mL of 0.2 mM DPPH methanolic solution was added to shoot and root extracts of squash landraces. Briefly 0.25 mM solution of DPPH radical (0.5 mL) was added to the sample solution in ethanol (1 mL) at a concentration (300 µg/ml). The reaction mixture was incubated in dark for 30 min and absorbance was measured at 517 nm using a UV-Vis spectrophotometer (Evolution 210, Thermo Scientific, Abingdon, UK). The antiradical activity was expressed as (%), which represent the concentration of the extract (mg/mL) required to inhibit 50% of the radicals.

### 2.5 Statistical analysis

Data were analyzed by a two-ANOVA according to the experimental design. The experimental design was *Completely Randomized Design* (CRD) with three replicates (Petri dishes). The effect of stress level was assessed across genotypes; the genotype performance was comparatively assessed across stress levels, while the landrace x stress level interaction effects were also determined. The significance of differences between pairs of means was assessed by the Duncan’s Multiple Range test (p< 0.05). Statistical analyses were performed using the Statistical Analysis System Software (v.9.0) (SAS Institute S.A., Cary, NC, USA).

## 3 Results

### 3.1 Response of squash landraces to drought stress at germination stage

Overall findings indicate that drought stress affected all traits related to germination and seedling growth, as evidenced by the mean values of GP, SVI, RL, RFW, SL, SFW, SL/RL and WU in controls and stressed plants. Further, the analysis of variance pointed as the most variable traits those directly linked to germination (WU, GP, SVI) (**Table 2**).

**Table 2:** Analysis of variance (mean squares) for traits related to seed germination and seedling growth of the studied squash landraces (accessions) under the different drought stress levels (0, -0.24, -.0.47 and - 0.73 MPa).

S.O.V.	DF	GP	SVI	RL	RFW	SL	SFW	SL/RL	WU
Accession	3	3269.87	59065.66	11.84	3.17	13.60	0.85	1.64	3919.46
Treatment	3	10103.12	10331613.85	89.52	0.39	363.27	1.24	3.18	5476.76
Accession x Treatment	9	561.09	211024.01	16.13	0.16	2.70	0.03	0.80	376.076
CV (%)	–	11.74	10.88	4.63	5.47	2.01	1.94	6.56	14.086

S.O.V., source of variance; CV, coefficient of variance; DF, degree of freedom; GP, germination percentage; SVI, seedling vigor index; RL, root length; RFW, root fresh weight; SL, shoot length; SFW, shoot fresh weight; SL/RL, ratio shoot length to root length; WU, seed water absorbance.

–, not applicable.

#### 3.1.1 Effect of the drought stress level across landraces

Seed germination varied significantly between the tested stress levels, while a decreasing trend was observed in GP. It is worth noting that at - 0.24 MPa and - 0.47 MPa squash landraces reached GP values >70%, indicating their high germination potential under drought stress conditions (**Supplementary Table S1**).

A similar trend was also recorded in seedling growth traits, with significantly decreased values at the highest level induced by D-mannitol (-0.73 MPa) (**Supplementary Table S1**). In particular, SL and RL were considerably affected by the stress level, showing values that ranged from 9.87 cm to 2.62 cm and from 6.82 cm to 3.04 cm in the control and at - 0.73 MPa, respectively. Accordingly, the shoot/root ratio (SL/RL) ranged from 1.53 to 0.91, indicating that shoot length was more profoundly affected than root length under drought stress conditions. Furthermore, at -0.47 MPa both SL and SFW were more pronouncedly affected than RL and RFW (decreased by 48.5% and 21.9%, compared to 37.9% and 16.2%, respectively), whereas at - 0.73 MPa only SL showed a higher decrease than RL (73.4% compared to 55.0%). Such values were also reflected in SVI, indicating that high drought stress significantly affects the overall growth of seedlings. Finally, RV was significantly decreased in stressed plants regardless of the stress level, as compared to the control treatment (11.40 cm^3^), although this decrease was proportional to the stress level applied (5.47 cm^3^ at - 0.73 MPa).

#### 3.1.2 Performance of landraces across stress levels

In terms of GP, landraces showed values ranging from 63.75% to 84.97%, indicating a variable response to drought stress (**Supplementary Table S2**). Landrace “751” showed the highest GP (84.97), thus providing evidence for its better adaptation to drought stress, whereas landrace “747” showed the lowest GP values (63.75%). Landraces “746” and “748” showed a mean GP of 79.41% and 69.69% respectively, suggesting a moderate drought tolerance. The superiority of landrace “751” was further depicted in SVI values, reflecting the rapidity of germination compared to other landraces. In contrast, the same landrace (“751”) presented the lowest values for SL (4.71 cm) and relatively low also for RL (4.61 cm), whereas the highest values for these parameters were recorded in landrace “747” (5.52 and 5.98 cm for RL and SL, respectively). In addition, drought stress had a more severe impact on root length than shoot length in the case of “746” and “747”, whereas landraces “748” and “751” showed the opposite trend. Significant differences were also observed for RFW and SFW, with “746” showing the highest value for both traits (**Supplementary Table S2**). Furthermore, landraces differed significantly in relation to WU, with “751” and “748” showing the highest and lowest values, respectively. Finally, the highest value of RV was noted in “746” landrace (11.54 cm^3^), whereas the lowest values were recorded in “748” (5.20 cm^3^) landrace.

#### 3.1.3 Landrace × drought stress levels interaction effects

The two-way ANOVA revealed that landraces differentially responded to varying stress levels, as evidenced by the respective interaction effects (landrace × drought stress levels) for all traits related to germination and seedling growth potential (**Table 3**). In untreated seeds, germination was considerably affected by the landrace, thus indicating their different innate germination potential, which is probably attributed to differences in the median longevity of seeds. Among landraces, “751” and “748” presented the highest and lowest GP under control conditions (97.77% and 85.77%, respectively), thus justifying that seed longevity is a genotype-dependent variable. Under stress conditions, the landraces differed significantly in relation to their germination potential at the different stress levels. As such, “751” ranked as the most tolerant at - 0.24 MPa and - 0.47 MPa (96.55% and 83.33% GP, respectively), whereas”747” was consistently the most sensitive genotype to all stress levels (68.33% and 33.33% GP). Interestingly, at - 0.73 MPa, “746” showed the highest GP (66.77%), followed by “751” (63.33%).

**Table 3:** Response of the studied squash landraces to different drought stress levels (0, -0.24, -.0.47 and - 0.73 MPa) in relation to germination and seedling growth traits (means ± SD).

Water potential (MPa)	Landrace	GP (%)	SVI (%)	RL (cm)	RFW (g)	SL (cm)	SFW (g)	SL/RL	WU	RV (cm^3^)
Control	“748”	85.77 ± 2.64^d*^	1776.88 ± 38.53^a^	8.55 ± 0.20^a^	0.67 ± 0.01^b^	9.77 ± 0.08^b^	0.84 ± 0.006^a^	1.14 ± 0.02^b^	88.77 ± 2.64^d^	9.35 ± 0.25^c^
	“751”	97.77 ± 1.44^a^	1287.58 ± 40.90^c^	6.66 ± 0.18^c^	0.03 ± 0.001^c^	9.68 ± 0.12^c^	0.63 ± 0.003^c^	1.45 ± 0.04^b^	96.66 ± 1.44^b^	12.38 ± 0.24^a^
	“747”	96.66 ± 2.22^b^	1581.41 ± 28.90^b^	7.64 ± 0.14^b^	0.01 ± 0.0004^d^	10.53 ± 0.06^a^	0.63 ± 0.003^c^	1.37 ± 0.02^b^	97.77 ± 2.22^a^	11.51 ± 0.34^b^
	“746”	92.22 ± 2.64^c^	1572.39 ± 50.82^b^	4.43 ± 0.22^d^	0.75 ± 0.02^a^	9.51 ± 0.03^d^	0.81 ± 0.005^b^	2.15 ± 0.10^a^	92.22 ± 2.64^c^	12.37 ± 0.37^a^
-0.24	“748”	74.11 ± 3.86^c^	856.27 ± 49.23^a^	5.35 ± 0.22^b^	0.56 ± 0.03^b^	5.29 ± 0.02^b^	0.62 ± 0.005^b^	0.98 ± 0.04^b^	74.11 ± 3.86^c^	7.44± 0.28^d^
	“751”	96.55 ± 1.05^a^	755.28 ± 20.76^d^	4.47 ± 0.21^c^	0.018 ± 0.0004^c^	3.59 ± 0.27^c^	0.38 ± 0.003^d^	0.80 ± 0.06^d^	96.55 ± 1.05^a^	9.33± 0.13^c^
	“747”	68.33 ± 3.90^d^	778.76 ± 9.76^c^	6.69 ± 0.17^a^	0.01 ± 0.0005^c^	5.83 ± 0.05^a^	0.40 ± 0.004^c^	0.87 ± 0.02^c^	68.33 ± 3.90^d^	9.56± 0.10^b^
	“746”	82.88 ± 1.73^b^	789.74 ± 43.48^b^	3.50 ± 0.23^d^	0.66 ± 0.01^a^	5.60 ± 0.05^a^	0.78 ± 0.01^a^	1.60 ± 0.10^a^	82.88 ± 1.73^b^	12.17 ± 0.33^a^
-0.47	“748”	68.88 ± 2.68^c^	548.13 ± 13.52^c^	3.30 ± 0.19^c^	0.15 ± 0.02^b^	2.90 ± 0.09^d^	0.41 ± 0.008^b^	0.88 ± 0.04^b^	68.88 ± 2.68^c^	2.53± 0.13^d^
	“751”	83.33 ± 4.63^a^	676.69 ± 13.85^a^	4.27 ± 0.21^b^	0.008 ± 0.0007^c^	3.39 ± 0.24^c^	0.22 ± 0.002^d^	0.79 ± 0.05^c^	83.33 ± 4.65^a^	7.46± 0.33^b^
	“747”	55.55 ± 1.30^d^	636.05 ± 29.70^b^	5.46 ± 0.25^a^	0.01 ± 0.0005^c^	4.40 ± 0.04^b^	0.35 ± 0.03^c^	0.80 ± 0.04^c^	55.55 ± 1.30^d^	6.43± 0.23^c^
	“746”	75.77 ± 1.35^b^	426.03 ± 12.77^d^	4.27 ± 0.16^b^	0.54 ± 0.006^a^	4.65 ± 0.08^a^	0.61 ± 0.003^a^	1.08 ± 0.03^a^	75.77 ± 1.35^b^	11.60 ± 0.19^a^
-0.73	“748”	50.00 ± 4.08^c^	183.54 ± 29.90^d^	2.43 ± 0.26^c^	0.07 ± 0.006^b^	1.87 ± 0.04^c^	0.31 ± 0.004^b^	0.77 ± 0.08^b^	50.00 ± 4.08^c^	1.46± 0.13^d^
	“751”	63.33 ± 1.44^b^	512.61 ± 13.53^a^	3.03 ± 0.15^b^	0.008 ± 0.0004^c^	2.17 ± 0.06^b^	0.18 ± 0.004^c^	0.71 ± 0.03^d^	63.33 ± 1.44^b^	6.47± 0.34^b^
	“747”	33.33 ± 5.27^d^	330.08 ± 9.11^b^	2.28 ± 0.27^c^	0.005 ± 0.0004^c^	3.18 ± 0.03^a^	0.17 ± 0.003^d^	1.40 ± 0.16^a^	33.33 ± 5.27^d^	4.28± 0.17^c^
	“746”	66.77 ± 1.47^a^	215.34 ± 18.59^c^	4.40 ± 0.37^a^	0.52 ± 0.006^a^	3.27 ± 0.05^a^	0.58 ± 0.006^a^	0.74 ± 0.05^c^	66.77 ± 1.47^a^	9.66± 0.21^a^

* Means in the same column and for the same D-mannitol concentration followed by the same letter are not significantly different at p< 0.05, according to Duncan’s Multiple Range test; GP, germination potential; SVI, seedling vigor index; RL, root length; RFW, root fresh weight; SL, shoot length; SFW, shoot fresh weight; SL/RL, ratio shoot length to root length; WU, seed water absorbance; RV, root volume.

The drastic effect of drought stress was further evidenced in both SVI and WU parameters, with landraces presenting varying responses to the different stress levels applied (**Table 3**). At both osmotic potentials of 0 MPa and - 0.24 MPa, “748”presented the highest values for SVI (1776.88% and 856.27%, respectively), whereas “751” showed the lowest values (1287.58% and 755.28% for 0 MPa and - 0.24 MPa, respectively). However, at higher stress levels, “751” was characterized by the highest SVI, while “746” and “748” were the most sensitive at - 0.47 MPa and - 0.73 MPa respectively. Similarly, “751” showed the highest WU values at -0.24 and - 0.47 MPa (96.55% and 83.33%, respectively), while at - 0.73 MPa the highest value was recorded in “746” (66.77%).

As far as traits related to seedling growth are concerned, the analysis also revealed significant interactions (p<0.001) among landraces and stress levels applied (**Table 3**). As expected, the response of all landraces to D-mannitol levels led a substantial reduction in both SL an RL. Although at lower stress levels “747”proved as most capable of retaining its growth ability, at -0.73 MPa “746”presented the highest values for both RL and SL, thus indicating its ability to withstand severe drought stress. In agreement with these data, the RFW and SFW were also drastically affected as D-mannitol concentration increased. The highest values for RFW were recorded in “746” both in controls and stressed plants, while for SFW the highest values were recorded in “748” and “746” in controls and stressed plants, respectively. In contrast, the lowest values were noted in “747” and “751” for both RFW and SFW. The SL/RL ratio was also profoundly affected by drought stress, especially at - 0.73 MPa, where landraces showed varying values ranging from 0.71 to 1.40. At this stress level, the highest SL/RL ratio was noted in “747”, reflecting the more drastic effect of water stress on roots than shoots (**Supplementary Table S1**). Evaluating the root volume (RV) at different D-mannitol levels, all landraces showed a decreasing trend, analogous to the stress level applied, thus proving the severe effect of drought stress on roots. In relation to RV, “746” and “748”presented the highest and lowest values at all stress levels applied, with the former being capable of retaining its root elongation ability even at - 0.73 MPa (9.66 cm^3^).

### 3.2 Physiological and biochemical response of squash landraces under drought stress at the plant stage

Our findings indicate that the response of the studied squash landraces to drought stress involved substantial changes occurring both at the physiological and biochemical level. As such, all traits related to chlorophyll fluorescence and contents of chlorophylls a and b and carotenoids, as well as real evapotranspiration and photosynthetic activity were significantly affected. The analysis further revealed significant changes in the accumulation of osmoprotectant compounds, such as MDA, free proline, phenolic compounds and flavonoids content and antioxidant activity assayed with DPPH method. It is worth noting, that among traits employed for evaluation of drought tolerance, chlorophyll b, F_m_, PA and the free proline content were the ones with the highest variability (**Table 4**).

**Table 4:** Analysis of variance (mean squares) for traits related to the physiological and biochemical response of squash landraces (accessions) to different drought stress levels ((0, -0.24, -.0.47 and - 0.73 MPa).

S.O.V.	DF	Chla	Chlb	Car	F_0_	F_m_	F_v_/F_m_	F_v_	PA	RET	MDA	Pro.	TPC.	TFC	DPPH
Accessions	5	115.09	34.75	0.16	44638.72	6272992.87	0.03	16.26	4187363.34	43148.17	74.86	4.22	648.98	1017.37	281.53
Treatment	3	309.53	68.95	0.83	195511.73	2761160.96	0.008	5.54	1950444.81	27821.72	264.29	16.54	1315.92	1812.89	995.07
Accession × Treatment	9	5.26^**^	10.92	0.01^**^	23261.41^**^	466898.21^**^	0.01^**^	3.29**	1773157.56^**^	2870.03^**^	50.01^**^	2.41^**^	77.87^**^	127.025^**^	47.97^**^
CV (%)	–	12.84	19.33	16.54	17.28	19.74	12.29	15.89	19.62	18.13	10.77	21.53	15.92	13.70	13.81

S.O.V., source of variance; DF, degree of freedom; CV, coefficient of variance; Chl a, chlorophyll a; Chl b, chlorophyll b; Car, carotenoids; F_0_, Minimal chlorophyll fluorescence intensity; F_m_, Maximum chlorophyll fluorescence intensity; F_v_/F_m_, Maximum quantum efficiency of PSII photosystem; F_v_, Variable chlorophyll fluorescence; PA, photosynthetic activity; RET, real evapotranspiration; MDA, Malondialdehyde; Pro, proline; TPC, Total phenolic compounds content; TFC, Total flavonoids content; DPPH, 1,1-diphenyl-2-picrylhydrazyl.

–, Not applicable; **, significantly different at p<0.01.

#### 3.2.1 Effect of drought stress on chlorophyll fluorescence parameters

Our findings indicate that the content of chlorophyll fluorescence parameters showed a decreasing trend at all stress levels, thus proving the adverse effects of drought stress (**Table 3**)

##### 3.2.1.1 Effect of drought stress across genotypes

D-mannitol concentrations induced changes on chlorophyll fluorescence, with the respective values differing significantly among stress levels (**Table 3**). At - 0.73 MPa, the minimum fluorescence (F_0_) and the variable fluorescence (F_v_) parameters showed the highest values (549.63 and 4.89, respectively) as compared to the controls (373.09 and 3.97, respectively). In contrast, the values of maximum fluorescence (F_m_) decreased as D-mannitol concentration increased (**Table 3**), leading to the highest decline at - 0.73 MPa (1722.97), whereas no significant differences were noted at -0.47 and - 0.24 MPa (1959.53 and 1948.96, respectively). Interestingly, the maximum efficiency of PSII, expressed by the ratio of F_v_/F_m_, showed a significant reduction at - 0.47 MPa, as compared to the control (0.780 and 0.800, respectively), while the respective values significantly increased at - 0.73 MPa (0.830) (**Table 3**).

##### 3.2.1.2 Performance of landraces across stress levels

Accordingly, the data underline the significance of landrace effect (*p*< 0.001) on the chlorophyll fluorescence parameters under drought stress conditions (**Table 4**). The variable response of the landraces to drought stress was evidenced for all fluorescence parameters, while the F_m_ was the most varied trait (CV > 19%) (**Table 4**).

Chlorophyll fluorescence was considerably affected by the landrace, as evidenced by the mean F_v_/F_m_ values (**Supplementary Table S4**), ranging from 0.76 to 0.82, with landraces “748”,and “747” showing the lowest and highest values, respectively, which are indicative of drought tolerance and drought susceptibility. Furthermore, all the landraces except for “748”, they could be characterized as drought tolerant since they showed mean F_v_/F_m_ values within the range of 0.79 - 0.82.

##### 3.2.1.3 Landrace x drought stress levels interaction effects

**Figure 1:**
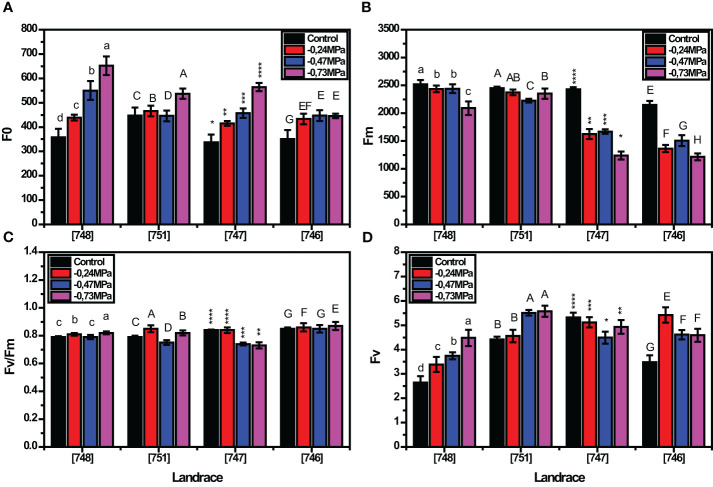
Response of squash landraces to different drought stress levels ((0, -0.24, -.0.47 and - 0.73 MPa) in relation to chlorophyll fluorescence parametersF_0_ **(A)**, F_m_ **(B)**, F_v_/F_m_ **(C)** and F_v_ **(D)**, 45 days after transplantation. Different letters above vertical bars of the same landrace indicate significant differences between the tested drought stress levels according to Duncan’s Multiple Range test at *p<* 0.05. Significance was indicated by small letter (a, b, c, d) for [748], by big letter (A, B, C, D) for[748], by stars for [747], and by big letters (E, F, G, H) for [746] landrace. The different number of asterisks (e.g., *, **, ***, ****) above the bars of genotype 747 indicate significant difference between the means of this genotype for each of the tested parameters.

As depicted in **Figure 1**, all the parameters related to chlorophyll fluorescence under drought stress conditions were variably affected by the landrace and the studied stress levels. In the absence of stress, F_0_, F_m_, F_v_ and F_v_/F_m_ were considerably affected by the landrace. Among landraces, “748” and “746” showed the lowest and the highest F_0_ values under control conditions (357.72 and 651.55, respectively; **Figure 1A**), while at the same time presenting the highest and the lowest F_m_ under the same conditions (2520.87 and 2089.11, respectively; **Figure 1B**). As expected, drought stress severely affected all chlorophyll fluorescence parameters for all landraces, with the severity of effects being increased with increasing stress intensity. At - 0.24 MPa, “746”, “747”and “751”, landraces were the most affected, representing a quantum ratio (F_v_/F_m_) of 0.85. Given that such values are outside the range of 0.79 - 0.82, their performance is classified as inferior in relation to stress tolerance. On the other hand, at -0.47 and - 0.73 MPa all the landraces presented a quantum ratio (F_v_/F_m_) outside of the mentioned range (**Figure 1C**) which indicates a significant effect of drought on the studied landraces. Furthermore, the response of most landraces to drought stress involved a drastic reduction in F_m_. In agreement with these findings, the F_v_ followed an increasing trend as D-mannitol concentration increased in landrace “751”, while the highest values for F_v_ were recorded in “746” both in control and - 0.24 MPa. As expected, the F_v_ was more profoundly affected at - 0.73 MPa with the landraces showing varying values ranging from 3.48 to 5.42 (**Figure 1D**).

#### 3.2.2 Effect of drought stress on the content of chlorophyll a, chlorophyll b and carotenoids

Similar to the effects of drought stress on chlorophyll fluorescence parameters, the content of pigments such as chlorophyll a and b and carotenoids, was drastically affected (**Supplementary Figure S1**).

##### 3.2.2.1 Effect of drought stress across genotypes

Chlorophyll a and carotenoids content decreased significantly as D-mannitol concentration increased. In particular, chlorophyll a values decreased from 13 mg g^-1^ FW in the control treatment to 6.41 mg g^-1^ FW in plants treated with - 0.73 MPa, while the respective decrease in carotenoids content was from 0.8 mg g^-1^ FWto 0.46 mg g^-1^ FW for the control treatment and the potential of - 0.73 MPa, respectively (**Supplementary Figure S1**). On the other hand, chlorophyll b presented a different trend where its content decreased only at high drought intensity (- 0.47 and 0.73 MPa) compared to the control treatment and the potential of - 0.24 MPa.

##### 3.2.2.2 Performance of landraces across drought stress levels

Our results revealed a significant effect of the landrace on the content of chlorophyll a, chlorophyll b and carotenoids under drought stress conditions (**Supplementary Table S5**). In relation to chlorophyll a and clorophyll b, the highest overall content was recorded in landrace “746” (11.24 mg g^-1^ FW and 4.94 mg g^-1^ FW, respectively), whereas the lowest value was recorded in “748”(7.48 mg g^-1^ FW and 2.67 mg g^-1^ FW). Regarding carotenoids, the highest content was recorded in landrace “751” (0.69 mg g^-1^ FW), while the lowest value was observed in landrace “748” (0.53 mg g^-1^ FW).

##### 3.2.2.3 Landrace × drought stress interaction effects

**Figure 2:**
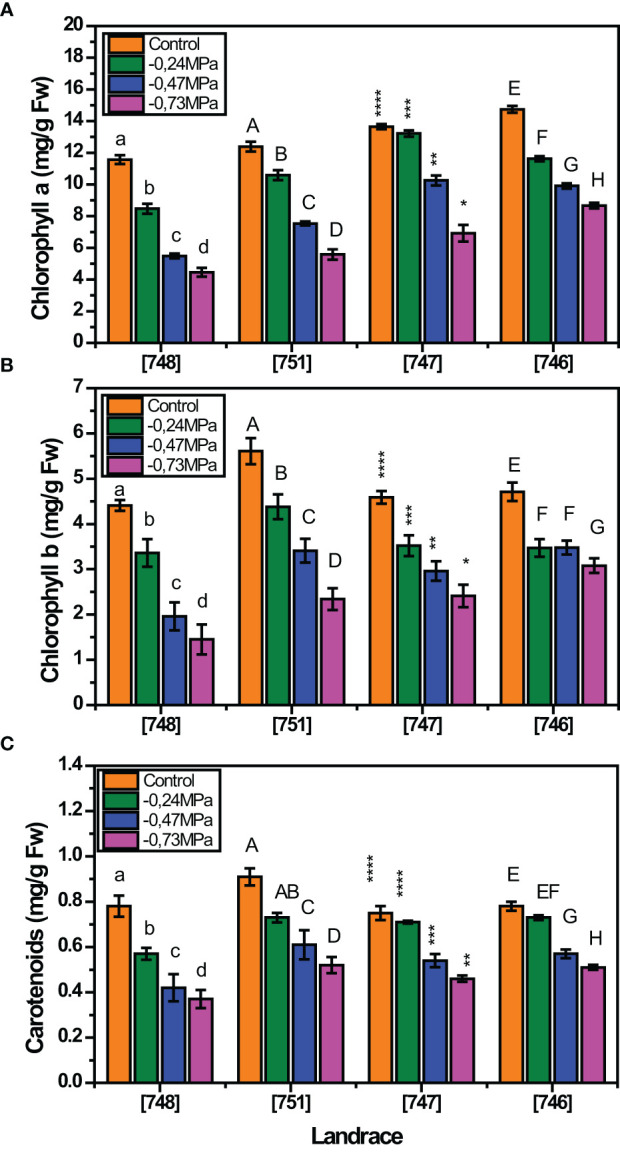
Response of squash landraces to different drought stress levels (0, -0.24, -0.47 and - 0.73 MPa) in relation to chlorophyll a **(A)**, chlorophyll b **(B)** and carotenoids **(C)**, measured at 45 days after transplantation. Different letters above vertical bars of the same landrace indicate significant differences between the tested drought stress levels according to Duncan’s Multiple Range test at *p<* 0.05. For [747] landrace, significance is indicated by the asterisk (*) symbol.

Our findings indicate that the contents of chlorophyll a, chlorophyll b and carotenoids were variably affected depending on the landrace and the stress level (**Figure 2**). In relation to chlorophyll a, all landraces showed a decreasing trend as D-mannitol increased (**Figure 2A**). A similar trend was recorded in the case of chlorophyll b, although “747” landrace seemed to be less affected with increasing severity of drought stress (**Figure 2B**). Furthermore, the content of carotenoids was differentially affected both by the landrace and the stress level, showing a general reduction in highly stressed plants (- 0.47 and 0.73 MPa) (**Figure 2C**).

#### 3.2.3 Effect of drought stress on real evapotranspiration and photosynthetic activity

##### 3.2.3.1 Effect of drought stress across genotypes

The real evapotranspiration (RET) and photosynthetic activity (PA) were evaluated on the 4^th^ fully expanded leaf, 45 days after transplantation. The analysis revealed the significant effects (*p*< 0.001, **Table 4**) of the studied drought stress levels on these variables (**Supplementary Figure S2**). Both RET and PAparameters recorded a significant reduction as D-mannitol concentrations increased. For RET, the stress effect was manifested through a decrease by 9.5%, 18% and 40% in plants subjected to -0.24, - 0.47 and - 0.73 MPa respectively, as compared to the control treatment (**Supplementary Figure S2A**). On the other hand, PA was less affected by drought stress, showing a 29% reduction at - 0.73 MPa, as compared to the controls, while no significant difference was noted between - 0.47 and - 0.73 MPa (**Supplementary Figure S2B**).

##### 3.2.3.2 Performance of landraces across drought stress levels

A significant effect of the landrace on both RET and PA parameters was also recorded (**Supplementary Table S6**). For RET, the highest value was noted in “746” landrace (179.44 mm/day), followed by “747” (137.99 mm/day), whereas “751” and”748”landraces showed lower values (104.08 and 108.94 mm/day, respectively) with no significant differences between them. Such findings are indicative of the differential response of landraces to drought stress, which is probably regulated through adjustments in stomatal closure. It is worth noting that the response of landraces to drought stress in relation to transpiration is similar to their photosynthetic response. Accordingly, the highest and lowest PA values were noted in landraces “746” and “751” respectively (1790.80 and 957.83 µmol photons m^-2^ s^-1^).

##### 3.2.3.3 Landrace × drought stress interaction effects

Our results further revealed significant interaction effects between landraces and drought stress levels, thus indicating the variability of the landraces under study to withstand varying drought stress levels (**Table 5**). All the tested landraces showed a general decreasing trend for both RET and PA parameters as D-mannitol concentration increased, indicating that stomatal closure which regulates photosynthesis by restring gas exchange between the atmosphere and the leaves is the first response when plants are subjected to drought stress. In relation to RET, all landraces were significantly affected by drought stress especially at high stress levels, yet their response varied considerably. As such, landraces “748” and “747”suffered the highest reduction, even at mild stress potential (e.g., - 0.24 MPa), whereas “751” and “746” were least affected since the reduction was more profound at potential higher than -0.47 MPa. For PA, a more pronounced decrease was noted at -0.47 and - 0.73 MPa for all the tested landraces. In particular, landraces “748” showed the better adaptability to high drought stress since RET values were reduced by 18.2% and 25.2% at - 0.47 and - 0.73 MPa, respectively. On the other hand, the rest of the landraces showed a similar response at - 0.47 MPa with reduction rates between 35.0% and 37.2%, while “751” landrace was the most severely affected at - 0.73 MPa showing a reduction of 75.7%.

**Table 5:** Response of squash landraces to different drought stress levels ((0, -0.24, -.0.47 and - 0.73 MPa) in relation to the real evapotranspiration and photosynthetic activity, measured at 45^th^ day after plantation.

Landrace	Water potential (MPa)	RET (mm H_2_O/day)	PA (µmol photons m^-2^ s^-2^)
“748”	Control	135.67 ± 3.00^a*^	1551.05 ± 22.86^a^
	-0.24	104.60 ± 2.37^b^	1328.61 ± 19.08^b^
	-0.47	101.55 ± 0.97^c^	1269.66 ± 26.67^c^
	-0.73	93.94 ± 2.85^d^	1160.16 ± 30.77^d^
“751”	Control	120.17 ± 40.16^b^	1471.88 ± 30.60^a^
	-0.24	125.00 ± 3.15^a^	1328.51 ± 19.08^b^
	-0.47	115.23 ± 2.98^c^	954.33 ± 27.01^c^
	-0.73	55.92 ± 2.95^d^	358.11 ± 25.80^d^
“747”	Control	189.99 ± 8.69^a^	1935.49 ± 34.63^a^
	-0.24	155.00 ± 3.09^b^	1838.33 ± 29.74^b^
	-0.47	131.42 ± 3.85^c^	1256.72 ± 25.27^c^
	-0.73	75.52 ± 2.82^d^	934.66 ± 25.20^d^
“746”	Control	194.52 ± 6.51^a^	1846.16 ± 30.49^a^
	-0.24	192.55 ± 3.05^b^	1659.49 ± 26.84^b^
	-0.47	176.61 ± 3.24^c^	1160.16 ± 30.77^c^
	-0.73	154.06 ± 2.39^d^	1080.17 ± 24.94^d^

* Means in the same column and for the same D-mannitol concentration followed by the same letter are not significantly different at p< 0.05, according to Duncan’s Multiple Range test; RET, real evapotranspiration; PA, photosynthetic activity.

#### 3.2.4 Effect of drought stress on MDA, free proline, total phenols, total flavonoids and DDDH scavenging activity

The content of osmoprotective compounds, such as MDA, free proline, total phenols, total flavonoids and DPPH, was significantly affected by D-mannitol-induced drought stress (**Supplementary Table S7**).

##### 3.2.4.1 Effect of drought stress across genotypes

At - 0.24 MPa, squash landraces presented a considerable increase in the content of MDA, free proline, total phenols, total flavonoids and DPPH, ranging to 23.27%, 63.33%, 16.13%, 16.24% and 19.69%, as compared to the control treatment, respectively (**Supplementary Table S7**). As expected, a further increase in concentration of D-mannitol at - 0.47 and - 0.73 MPa induced the accumulation of osmoprotectants, thus leading to significantly increased values for all the tested parameters, except for the case of MDA where no significant increase was observed at potential higher than -0.47 MPa. In particular, at - 0.47 MPa, the increase in the content of MDA, free proline, total phenols, total flavonoids and DPPH ranged to 39.77%, 86.66%, 25.60%, 26.02% and 28.54%, as compared to the control treatment, whereas at - 0.73 MPa the respective contents increased by 41.18%, 127.77%, 31.77%, 27.62% and 32.27%, over the control (**Supplementary Table S7**).

##### 3.2.4.2 Performance of landraces across drought stress levels

The results presented in **Supplementary Table S8** indicate a significant effect of the landrace on the content of MDA, free proline, total phenols, total flavonoids and DPPH. In relation to MDA, the highest content was recorded in landrace “747” (14.08 µmol g^-1^ FW) without being significantly different from landrace “746”, whereas the lowest value was recorded in landrace “748”. On the other hand, landrace “746” recorded the highest content of free proline (1.86 µg mg^-1^ FW), while the lowest respective values were noted in landraces “747” and “751” (1.32 and 1.38 µg mg^-1^ FW, respectively). Regarding TP and TF content and DPPH, the lowest values were detected in landrace “751” (33.28 mg GA/100 mg DW, 42.36 mg QE/100 mg DW and 26.64%, respectively), whereas “748” was the landrace with the highest overall content in phenolic compounds and the antioxidant activity (40.42 mg GA/100 mg DW, 51.78 mg QE/100 mg DW and 31.38%, respectively).

##### 3.2.4.3 Landrace × drought stress interaction effects

From a breeding perspective, our findings indicate the existence of significant genotype *×* drought stress interaction effects in relation to the content of osmoprotectants (**Table 6**). In relation to MDA content, landraces “751”, “747”and “746”showed an increasing trend as D-mannitol concentration increased, whereas MDA content in “748” landrace showed fluctuating values with a notable increase at - 0.24 and - 0.47 MPa and a decrease at - 0.73 MPa. On the other hand, all the landraces showed an increased content over the control treatment when D-mannitol concentration increased, especially in landraces “746” and”748” where a gradual increase with increasing content of D-mannitol was recorded. Similar trends were detected in the case of TP and TF content which also showed a gradual increase with increasing D-mannitol content. Moreover, it has to be mentioned the pronounced increase in TP and TF over the control for “748”, especially at - 0.73 MPa where TP and TF content increased by 207.7% and 41.2%, respectively. This particular trend was also confirmed in the case of DPPH antioxidant activity where the highest activity was recorded at the highest potential (-0.73 MPa) for all the tested landraces.

**Table 6:** Response of squash landraces to different drought stress levels ((0, -0.24, -.0.47 and - 0.73 MPa) in relation to the content of malondialdhehyde (MDA), free proline (FP), total phenolic compounds (TP), total flavonoids (TF) and DPPH activity (means ± SD).

Landrace	Water potential (MPa)	MDA (µmol g^-1^ FW)	Free proline (µg mg^-1^ FW)	TP (mg GA/100 mg DW)	TF (mg QE/100 mg DW)	DPPH (%)
“748”	Control	11.00 ± 0.48^c*^	0.64 ± 0.14^d^	30.97 ± 0.17^d^	40.15 ± 0.43^d^	22.90 ± 1.04^d^
	-0.24	12.23 ± 0.34^b^	1.45 ± 0.29^c^	41.59 ± 1.22^c^	54.29 ± 0.94^c^	32.48 ± 1.73^c^
	-0.47	13.04 ± 1.49^a^	1.79 ± 0.19^b^	43.18 ± 0.53^b^	55.96 ± 0.57^b^	34.62 ± 1.30^b^
	-0.73	10.17 ± 1.00^d^	2.29 ± 0.31^a^	95.31 ± 2.13^a^	56.71 ± 0.56^a^	35.52 ± 1.31^a^
“751”	Control	9.83 ± 0.71^d^	0.66 ± 0.07^d^	28.09 ± 1.88^d^	39.22 ± 0.82^c^	21.17 ± 1.94^d^
	-0.24	12.54 ± 0.93^c^	1.77 ± 0.16^b^	33.03 ± 2.59^c^	42.15 ± 0.89^b^	26.89 ± 1.63^c^
	-0.47	13.96 ± 1.45^b^	1.82 ± 0.14^a^	34.90 ± 3.53^b^	44.26 ± 0.99^a^	28.85 ± 1.51^b^
	-0.73	17.11 ± 1.05^a^	1.46 ± 0.17^c^	37.10 ± 3.25^a^	44.83 ± 0.93^a^	29.64 ± 1.54^a^
“747”	Control	10.78 ± 0.14^d^	1.15 ± 0.42^d^	32.33 ± 1.03^d^	39.19 ± 1.45^d^	24.21 ± 0.98^c^
	-0.24	14.42 ± 1.37^c^	1.67 ± 0.25^a^	35.24 ± 1.15^c^	45.89 ± 1.69^c^	27.30 ± 1.04^b^
	-0.47	15.27 ± 1.16^b^	1.48 ± 0.10^c^	41.48 ± 1.07^b^	50.84 ± 1.51^b^	30.35 ± 0.85^a^
	-0.73	15.86 ± 1.84^a^	1.57 ± 0.13^b^	42.03 ± 1.01^a^	51.41 ± 1.45^a^	30.84 ± 0.60^a^
“746”	Control	10.83 ± 0.09^d^	1.15 ± 0.12^d^	33.29 ± 1.26^d^	41.38 ± 1.26^c^	25.55 ± 1.41^d^
	-0.24	12.41 ± 1.12^c^	1.59 ± 0.27^c^	34.91 ± 1.73^c^	43.21 ± 1.41^b^	28.03 ± 1.68^c^
	-0.47	15.65 ± 1.42^b^	1.81 ± 0.11^b^	36.43 ± 1.74^b^	50.02 ± 1.62^a^	29.36 ± 1.16^b^
	-0.73	16.16 ± 1.88^a^	2.87 ± 0.28^a^	39.39 ± 1.05^a^	50.62 ± 1.33^a^	31.18 ± 1.36^a^

* Means in the same column and for the same D-mannitol concentration followed by the same letter are not significantly different at p< 0.05, according to Duncan’s Multiple Range test.

#### 3.2.5 Variation in the contents of MDA, FP, TP, TF and DPPH activity in the shoots and the roots of squash landraces subjected to drought stress

Significant differences in the content of osmoprotectants were also recorded between the roots and shoots (**Table 7**). Specifically, roots showed an increased content of MDA, free proline, TF and DPPH as compared to the shoots (13.78 *vs* 12.71 µmol g^-1^ FW, 2.10 *vs* 0.95 µg mg^-1^ FW, 47.20 *vs* 46.56 mg QE/100 mg DW and 28.98% *vs* 28.38%, respectively), whereas only TP content was higher in the shoots than roots (37.57 *vs* 36.22 mg GA/100 mg DW, respectively).

**Table 7:** Mean effect of shoot and root tissue on the content of MDA, FP, TP, TF and DPPH activity.

Organ	MDA (µmol g^-1^ FW)	Free proline (µg mg^-1^ FW)	TP (mg GA/100 mg DW)	TF (mg QE/100 mg DW)	DPPH (%)
Shoots	12.71_b_*	0.95^b^	37.57^a^	46.56^b^	28.38^b^
Roots	13.78^a^	2.10^a^	36.22^b^	47.20^a^	28.98^a^

^*^ Means in the same column followed by the same letter are not significantly different at p< 0.05, according to Duncan’s Multiple Range test; MDA: Malondialdhehyde; TP: Total phenols; TF: Total flavonoids; DPPH: 2,2-diphényl 1-picrylhydrazyle.

**Figure 3:**
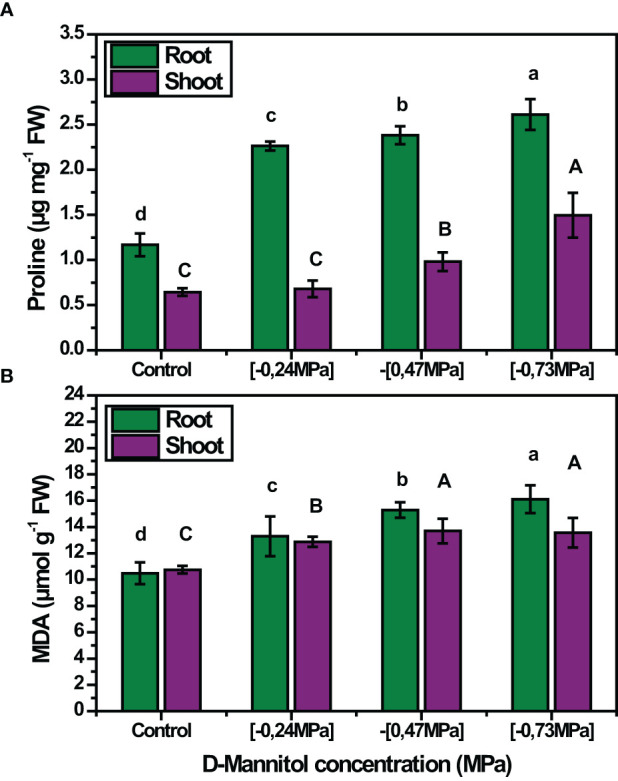
Mean effect of drought stress level (0, -0.24, -0.47 and - 0.73 MPa) on proline content **(A)** and on MDA **(B)** of squash landraces. Different letters above the bars (shoots: A, B, C, D and roots: a, b, c, d) indicate significant differences according to Duncan Multiple Range test at *p<* 0.05.

The accumulation of MDA was also affected by the stress level applied, with its content being proportional to D-mannitol concentration in both roots and shoots (**Figure 3A**). In roots, the MDA content ranged from 10.47 to 16.10 µmol g^-1^ FW at 0 mM and - 0.73 MPa respectively, while in shoots the values ranged from 10.75 to 13.55 µmol g^-1^ FW. It is worth noting that in all cases stressed plants showed a higher MDA content in roots than in shoots. A similar trend was also noted for free proline content which was maximum at - 0.73 MPa in both roots and shoots, corresponding to an increase of 125% and 132.81% compared to the control treatment, respectively (**Figure 3B**).

**Figure 4:**
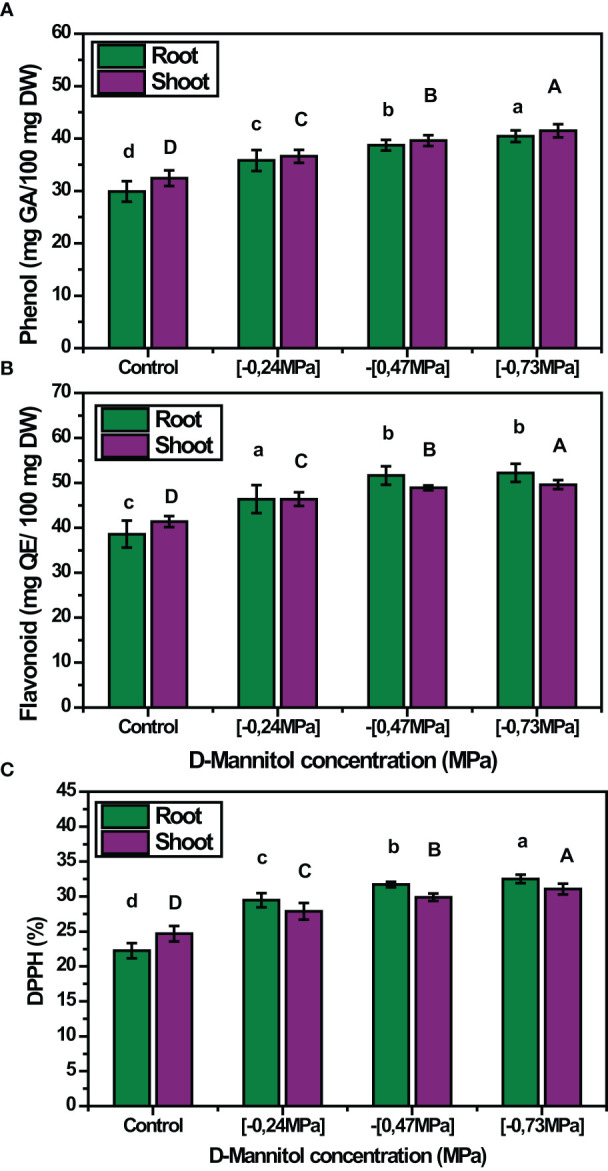
Mean effect of drought stress level (0, -0.24, -0.47 and - 0.73 MPa) **(A)** on total phenolic compounds content (mg GA 100 g^-1^ FW); **(B)** total flavonoids content (mg QE 100 g^-1^ FW); and **(C)** DPPH activity (%) of the studied squash landraces. Different letters above the bars (shoots: A, B, C, D; and roots: a, b, c, d) indicate significant differences according to Duncan Multiple Range test at *p<* 0.05.

Moreover, drought stress caused an increase in the contents of total phenolic compounds (TP), total flavonoids (TF) and DPPH in both shoots and roots of squash landraces (**Figures 4A–C**). The content of phenolics was proportional to the stress level applied, thus showing a gradual increase in both shoots and roots (- 0.24 MPa: 12.79% and 19.73%, - 0.47 MPa: 22% and 29.53%, - 0.73 MPa: 27.83% and 35.25% increase over the control treatment in shoots and roots, respectively), as D-mannitol concertation increased (**Figure 4A**). A similar trend was also observed for total flavonoids (TF), whose content presented a significant increase in both shoots and roots (- 0.47 MPa: 18.06% and 33.94%, -0.73 MPa: 19.75% and 35.42% increase over the control treatment in shoots and roots, respectively) (**Figure 4B**). Finally, drought stress induced a considerable increase in DPPH contents, compared with the respective control, which ranged between 24.68% and 31.08% and from 22.24% to 32.52% in shoots and roots, respectively (**Figure 4C**).

## 4 Discussion

The results of the present study underline the drastic effects of drought stress depending on the landrace, the stress level as well as their interaction. During germination, all traits related to both germination ability and rate (GP, WU, SVI), as well as seedling growth (RL, SL, RFW, SFW, RV) were negatively affected in all the studied landraces, with the effects being proportional to the stress level applied, thus providing evidence for their relative sensitivity or tolerance to drought stress. Such findings are further supportive of previous reports related to the severe effects of drought stress during germination in a plethora of crop species, including maize (Queiroz et al., 2019), sorghum (Cokkizgin et al., 2019), faba bean (Mombeini et al., 2021), cucumber (Rahmani et al., 2019), as well as pumpkin (*Cucurbita pepo* L.; Biareh et al., 2022). The drought stress effects during germination are widely attributed to the fact that this stage is considered as the most sensitive throughout the plant life cycle since water uptake, referred to as imbibition, is a prerequisite to initiate germination. Accordingly, the amount of the absorbed water depends both on the level of the initial seed moisture content, as well as on its chemical composition (McDonald et al., 1988). In fact, protein and pectin are hydrophilic colloids, thus requiring more water than starch to promote imbibition and germination processes (Queiroz et al., 2019). In addition to germination potential, our findings further pointed to the drastic effects of drought stress on seedling growth, with the increasing concentration of D-mannitol leading to gradually decreased elongation in both roots and shoots. Such seedling growth inhibitory effects have been also reported in a wide range of plant species and are mainly attributed to osmotic stress effects (Queiroz et al., 2019). In our study, the findings which suggest that roots were more drastically affected than shoots are in agreement with previous reports which also suggest that the aboveground parts are more affected than the aerial parts in several species when the plant is subjected to drought stress, broad been (Cokkizgin et al., 2019) and tomato plants (Al Hassan et al., 2015), but also in squash germplasm under salt stress conditions (Tarchoun et al., 2022). Despite the fact that drought stress posed a limiting factor in germination and growth potential of squash landraces, it is worth noting that the tested landraces showed a differential response to varying stress levels. Among landraces, “751” and”746”exhibited a higher germination potential at all stress levels, thus indicating their superiority in terms of drought tolerance. Given that these particular landraces were capable of germination and seedling growth even under severe stress conditions (-0.47 and - 0.73 MPa), it could be assumed that they consist a promising genetic material to be further exploited for breeding purposes.

Furthermore, our data underlined the significant effect of drought stress in all the studied physiological and biochemical traits, since chlorophyll b, F_m_, PA and free proline content were the most variable traits, thus providing evidence for their suitability as screening criteria for drought tolerance among the tested landraces. Chlorophyll fluorescence analysis, which is routinely employed to assess the status of plant photosynthesis, revealed significant variations not only for the landrace and the drought stress level but also for their interactions. Changes in the minimum fluorescence yield (F_O_) values suggest differential response to the transfer of excitation energy between pigments molecules (Liang et al., 2020). In our study, maximum fluorescence (F_m_), whose values are maximum in healthy non-stressed leaves that have been dark-adapted (Huang et al., 2019), showed a decreasing trend as D-mannitol concentration increased. Furthermore, the F_v_/F_m_ ratio, which refers to the maximum quantum yield of PSII photosynthetic apparatus and is commonly used as indicator of stress sensitivity (Shin et al., 2021), presented the lowest value at - 0.47 MPa (0.78), thus reflecting the existence of stress which lead to inactivation damage of PSII, referred to as photoinhibition. However, the F/Fm ratio showed higher values at - 0.73 MPa (0.83) which are indicative of unstressed leaves. Similar drought stress effects in photosynthesis activity were also reported in other plant species, including lettuce (Inoue et al., 2021), tomato and pepper (Parkash and Singh, 2020). According to previous studies, such disturbances due to abiotic stress, including water deficit, are attributed to changes in the activity of pigments in the course of photochemical and enzymatic reactions in thylakoids and the chloroplast, resulting in low amount of energy absorbed by PSII, as well as in changes in the membrane permeability of chloroplasts (Wang et al., 2018). Apart from the obvious stress level effects, our data pointed to differences in chlorophyll fluorescence attributed to the effect of landrace. Interestingly, landraces “747” and “746” showed a notable decrease at - 0.47 MPa, which is indicative of a stress state as a result of the initial plant damage closely related to PSII, whereas “751” and “748”exhibited an increased F_v_/F_m_ ratio at both - 0.47 and - 0.73 MPa.

Moreover, our results suggested the drastic effect of drought stress on the content of chlorophylls a, b and carotenoids as well as on real evapotranspiration (RET) and photosynthetic activity (PA), while it was evidenced that the stress effects were analogous to its intensity. Our findings also confirm that the decreased content of chlorophylls and carotenoids, coupled with the decreased values for RET and PA, reflect the first defense reaction of leaves, involving stomatal closure as a means to reduce transpiration and photosynthesis as well as to limit CO_2_ exchange, as previously reported in other vegetable crops (Parkash and Singh, 2020). Although the observed reduction in RET and PA values under D-mannitol stress conditions is in accordance with previous reports in other plant species, such as s, cucumber (Li et al., 2008) and watermelon (Mo et al., 2016), it is worth noting that landrace “746”, despite of its high RET values, also exhibited the highest PA values (1790.84 µmol photons m^-^² s^-1^) at - 0.73 MPa, thus reflecting an ability to maintain its photosynthetic activity even under severe drought stress. In contrast, landrace “751” was characterized by the lowest values for both RET and PA, probably as a result of adjustments in stomatal closure. Such regulation of stomatal control under drought stress conditions consists the main physiological factor in the context of optimizing water use and preventing excessive water loss, as it has already been proven in soybean (Liu et al., 2005). In the same line, it has been evidenced that wheat’s response to drought stress involved a decreased rate of photosynthesis and transpiration as well as decreased stomatal conductance (decrease by 78.4%, 85.4% and 92%, respectively), the latter being more correlated with the transpiration rate than the photosynthetic rate (Farooq et al., 2014).

It is well evidenced that plants’ response to abiotic stress involves the enhanced accumulation of osmolytes, such as free proline, MDA, total phenols and flavonoids, as well as increased radical-scavenging activity to mitigate oxidative stress (Kapoor et al., 2020; Parkash and Singh, 2020; Yadav et al., 2021). Among compounds with osmoprotective function, free proline is one of the most well studied indicators for drought tolerance as it plays a pivotal role in regulating osmotic pressure in stressed plants (Yadav et al., 2021). Similarly, MDA generated by the peroxidation of polyunsaturated fatty acids of the cell membranes as a response to ROS generation (Zhou et al., 2017), is routinely employed as an indicator to evaluate the degree of plasma membrane damage and the ability of plants to cope with drought stress. In relation to both free proline and MDA, our findings indicate a differential response of roots and shoots, with the former showing an increased content thus suggesting more intense stress conditions. Such observations are in agreement with the fact that under drought stress conditions roots play a fundamental role both in the perception of water deficit status and hormone signal transduction, mediated by abscisic acid (ABA), the latter triggering signals to aerial organs and tissues through vascular bundles, causing morphological/anatomical alterations, such as the root to shoot ratio (Takahashi et al., 2020). As the first organ to drought perception, roots respond with a series of morphological changes in the growth and architecture, including their length, weight and volume, while, at the same time, constituting the primary site of osmolyte accumulation (Fang and Xiong, 2015). Such drought adaption mechanisms in roots regulate water loss and promote water use efficiency, which are considered as key functional traits that contribute to drought tolerance (Takahashi et al., 2020). In accordance with the drought responses in typical roots, our findings suggest a more pronounced increase in total phenolic compounds and total flavonoids content as well as in DPPH activity in roots than in shoots, especially at high stress levels, which is indicative of the essential role of these parameters in mitigating the water deficit effects.

## 5 Conclusions

Conclusively, our findings contribute towards understanding the response of squash landraces to drought stress and further provide evidence regarding the tolerance ability of the landraces under study. Among landraces, “751” and”746”were classified as better performing in terms of drought tolerance both at germination and later plant growth stages. Considering the small number of landraces under study, it is concluded that squash germplasm exhibits considerable variation for drought tolerance traits, thus providing ground both for selecting appropriate landraces for cultivation under water deficit conditions and applying breeding approaches targeted at improving crop’s drought tolerance. However, further studies are needed with a broader group of germplasm accessions of squash germplasm that will help to valorize the existing variability through the selection of elite genotypes in targeted breeding programs.

## Data availability statement

The raw data supporting the conclusions of this article will be made available by the authors, without undue reservation.

## Author contributions

WA: methodology; formal analysis; investigation; data curation; NT: conceptualization; writing-original draft preparation; writing-review and editing; visualization; supervision; project administration; IM and RA: methodology; formal analysis; investigation; data curation; OP: writing-original draft preparation; HF: methodology; formal analysis; investigation; data curation; CA: methodology; formal analysis; investigation; data curation; RK: writing-review and editing; visualization; supervision; project administration; SAP: conceptualization; writing-review and editing; visualization; supervision; project administration; funding acquisition. All authors contributed to the article and approved the submitted version.
