# Supplementary material for: Effects of drought stress induced by D-Mannitol on the germination and early seedling growth traits, physiological parameters and phytochemicals content of Tunisian squash (Cucurbita maximaDuch.) landraces
Source: Front Plant Sci. 2023 Jul 31;14:1215394. doi: 10.3389/fpls.2023.1215394 (PMC10432687; doi:10.3389/fpls.2023.1215394)
Supplement: Supplementary file 1 [file DataSheet_1.pdf]

Table S1. Mean effect of the drought stress level (0, 100, 200, 300 mM of D-mannitol) on traits related to germination and seedling growth of the studied squash landraces.

**D-mannitol concentration (mM)**	**GP (%)**	**SVI (%)**	**RL (cm)**	**RFW (g)**	**SL (cm)**	**SFW (g)**	**SL/RL**	**WU (%)**	**RV (cm^3)**
Control	93.11^{a}	1554.7^{a}	6.82^{a}	0.37^{a}	9.87^{a}	0.73^{a}	1.53^{a}	83.25^{a}	11.40^{a}
100	80.47^{b}	795.02^{b}	4.23^{b}	0.31^{b}	5.08^{b}	0.57^{b}	1.07^{b}	73.25^{b}	9.71^{b}
200	70.88^{c}	571.73^{c}	3.33^{c}	0.18^{c}	3.83^{c}	0.26^{d}	0.89^{c}	65.30^{c}	7.00^{c}
300	53.36^{d}	310.40^{d}	3.04^{d}	0.15^{d}	2.62^{d}	0.31^{c}	0.91^{c}	54.14^{d}	5.47^{d}
F-Value	132.01**	1334.29**	1820.98**	2026.96**	3164.00**	1237.00**	608.79**	688.00**	46.32**

** Means in the same column followed by the same letter are not significantly different at $p < 0.05$, according to Duncan's Multiple Range test; GP: germination potential, SVI: seedling vigor index; RL: root length; RFW: root fresh weight; SL: shoot length; SFW: shoot fresh weight; SL/RL: ratio shoot length to root length; WU: seed water absorbance, RV: root volume

Table S2. Mean effect of the studied squash landraces on traits related to germination and seedling growth under the different drought stress levels (0, 100, 200, 300 mM of D-mannitol).

**Landrace**	**GP (%)**	**SVI (%)**	**RL (cm)**	**RFW (g)**	**SL (cm)**	**SFW (g)**	**SL/RL**	**WU (%)**	**RV (cm^3)**
"748"	69.69^c	750.88^d	4.91^b	0.36^b	4.96^c	0.55^b	0.94^c	58.11^d	5.20^d
"751"	84.97^a	831.58^a	4.61^c	0.01^c	4.71^d	0.35^d	0.94^c	80.44^a	8.91^b
"747"	63.75^d	821.21^b	5.52^a	0.01^c	5.98^a	0.41^c	1.11^b	62.50^c	7.95^c
"746"	79.41^b	808.05^c	4.15^d	0.62^a	5.76^b	0.70^a	1.39^a	74.89^b	11.54^a
F-Value	42.72**	7.63*	239.57**	16221.4**	1167.40**	239.57**	315.23**	423.18**	4483.01**

** Means in the same column followed by the same letter are not significantly different at $p < 0.05$, according to Duncan’s Multiple Range test; GP: germination potential, SVI: seedling vigor index; RL: root length; RFW: root fresh weight; SL: shoot length; SFW: shoot fresh weight; SL/RL: ratio shoot length to root length; WU: seed water absorbance; RV: root volume

Table S3. Mean effect of the drought stress level (0, 100, 200, 300 mM D-mannitol on chlorophyll fluorescence F_0, F_m, Fv and F_v/F_m evaluated 45 days after transplantation

	F_0	F_m	F_v	F_v/F_m
Control	$373.09^{d} \pm 19.35$	$2386.25^{a} \pm 59.09$	$3.97^{c} \pm 1.05$	$0.80^{b} \pm 0.04$
100 mM	$437.96^{c} \pm 16.23$	$1948.96^{b} \pm 86.03$	$4.62^{b} \pm 0.81$	$0.81^{b} \pm 0.04$
200 mM	$474.97^{b} \pm 18.80$	$1959.53^{b} \pm 74.81$	$5.59^{a} \pm 0.68$	$0.78^{c} \pm 0.05$
300 mM	$549.63^{a} \pm 23.42$	$1727.97^{c} \pm 86.83$	$4.89^{b} \pm 0.52$	$0.83^{a} \pm 0.05$

Table S4. Mean effect of squash landraces on traits related to chlorophyll fluorescence under the different drought stress levels (0, 100, 200, 300 mM of D-mannitol).

**Landrace**	$\mathbf{F_0}$	$\mathbf{F_m}$	$\mathbf{F_v/F_m}$	$\mathbf{F_v}$
"748"	499.38$^{\mathbf{d}}$	1738.54$^{\mathbf{c}}$	0.76$^{\mathbf{c}}$	3.57$^{\mathbf{c}}$
"751"	474.22$^{\mathbf{c}}$	1569.54$^{\mathbf{d}}$	0.81$^{\mathbf{ab}}$	5.02$^{\mathbf{a}}$
"747"	443.10$^{\mathbf{b}}$	2420.15$^{\mathbf{a}}$	0.82$^{\mathbf{a}}$	4.97$^{\mathbf{a}}$
"746"	418.94$^{\mathbf{a}}$	2294.63$^{\mathbf{b}}$	0.80$^{\mathbf{b}}$	4.53$^{\mathbf{b}}$
F-Value	39.97**	164.51**	90.77**	229.10**

**Means in the same column followed by the same letter are not significantly different at $p < 0.05$, according to Duncan's Multiple Range test; F_0: Minimum chlorophyll fluorescence intensity; F_m: Maximum chlorophyll fluorescence intensity; F_v/F_m: Maximum quantum efficiency of PSII photosystem; F_v: Variable chlorophyll fluorescence.

Table S5. Mean effect of squash landraces on chlorophyll a, chlorophyll b and carotenoids under the different drought stress levels (0, 100, 200, 300 mM D-mannitol).

**Landrace**	**Chlorophyll a**	**Chlorophyll b**	**Carotenoids**
"748"	7.48^{d}	2.67^{b}	0.53^{d}
"751"	9.03^{c}	3.94^{ab}	0.69^{a}
"747"	11.06^{b}	3.20^{b}	0.62^{c}
"746"	11.24^{a}	4.94^{a}	0.65^{b}
F-Value	1505.76^{**}	3.06^{*}	96.85^{**}

** Means in the same column followed by the same letter are not significantly different at $p < 0.05$, according to Duncan's Multiple Range test.

Table S6. Mean effect of squash landraces on real evapotranspiration and photosynthetic activity under the different drought stress levels (0, 100, 200, 300 mM of D-mannitol).

**Landrace**	**RET (mm/day)**	**PA (µmol m^{-2} s^{-1})**
"748"	108.944^{c}	1327.375^{c}
"751"	104.084^{c}	957.833^{d}
"747"	137.986^{b}	1341.305^{b}
"746"	179.439^{a}	1790.840^{a}
F-Value	370.74^{**}	5923.84^{**}

** Means in the same column followed by the same letter are not significantly different at $p < 0.05$, according to Duncan's Multiple Range test; RET: evapotranspiration; PA: photosynthetically active radiation.

Table S7. Mean effect of the drought stress level (0, 100, 200, 300 mM of D-mannitol) on the content of osmoprotective compounds (MDA, FP, TP, TF and DPPH).

**D-mannitol concentration (mM)**	**MDA (µmol g^{-1} FW)**	**Free proline (µg mg^{-1} FW)**	**TP (mg GA/100 mg DW)**	**TF (mg QE/ 100 mg DW)**	**DPPH (%)**
Control	10.61^{c}	0.90^{d}	31.17^{d}	39.89^{d}	23.96^{d}
100	13.08^{b}	1.47^{c}	36.20^{c}	46.37^{c}	28.68^{c}
200	14.83^{a}	1.68^{b}	39.15^{b}	50.27^{b}	30.80^{b}
300	14.98^{a}	2.05^{a}	40.95^{a}	50.91^{a}	31.80^{a}
F-Value	129.55	152.75	275.56	601.36	828.67

[**] Means in the same column followed by the same letter are not significantly different at $p < 0.05$, according to Duncan's Multiple Range test; MDA: Malondialdhehyde; TP: Total phenols; TF: Total flavonoids; DPPH: 2,2-diphényl 1-picrylhydrazyle.

Table S8. Mean effect of squash landraces on the content of osmoprotectant compounds (MDA, FP, TP, TF and DPPH) under the different drought stress levels (0, 100, 200, 300 mM of D-mannitol).

**Landrace**	**MDA (µmol g^{-1} FW)**	**Free proline (µg mg^{-1} FW)**	**TP (mg GA/100 mg DW)**	**TF (mg QE/ 100 mg DW)**	**DPPH (%)**
"748"	11.78^{c}	1.54^{b}	40.42^{a}	51.78^{a}	31.38^{a}
"751"	13.36^{b}	1.38^{c}	33.28^{d}	42.36^{c}	26.64^{c}
"747"	14.08^{a}	1.32^{c}	37.77^{b}	46.81^{b}	28.18^{b}
"746"	13.76^{ab}	1.86^{a}	36.00^{c}	46.31^{b}	28.53^{b}
F-Value	36.70	38.98	135.90	337.47	234.46

** Means in the same column followed by the same letter are not significantly different at $p < 0.05$, according to Duncan's Multiple Range test; MDA: Malondialdhehyde; TP: Total phenols; TF: Total flavonoids; DPPH: 2,2-diphényl 1-picrylhydrazyle; GA: Gallic acid; QE: Quercetin.

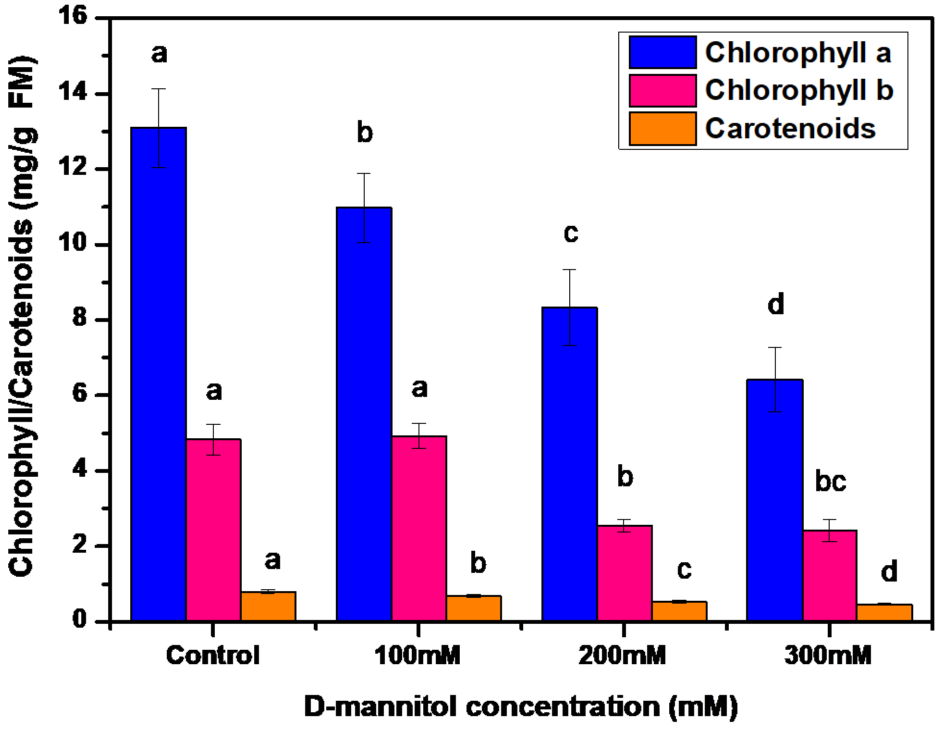

Figure S1. Mean effect of the studied drought stress levels (0, 100, 200, 300 mM of D-mannitol) on chlorophyll a (A), chlorophyll b (B), and carotenoids (C) content, measured at 45 days after transplantation. Different letters above the bars indicate significant difference at $p < 0.05$, according to Duncan's Multiple Range test.

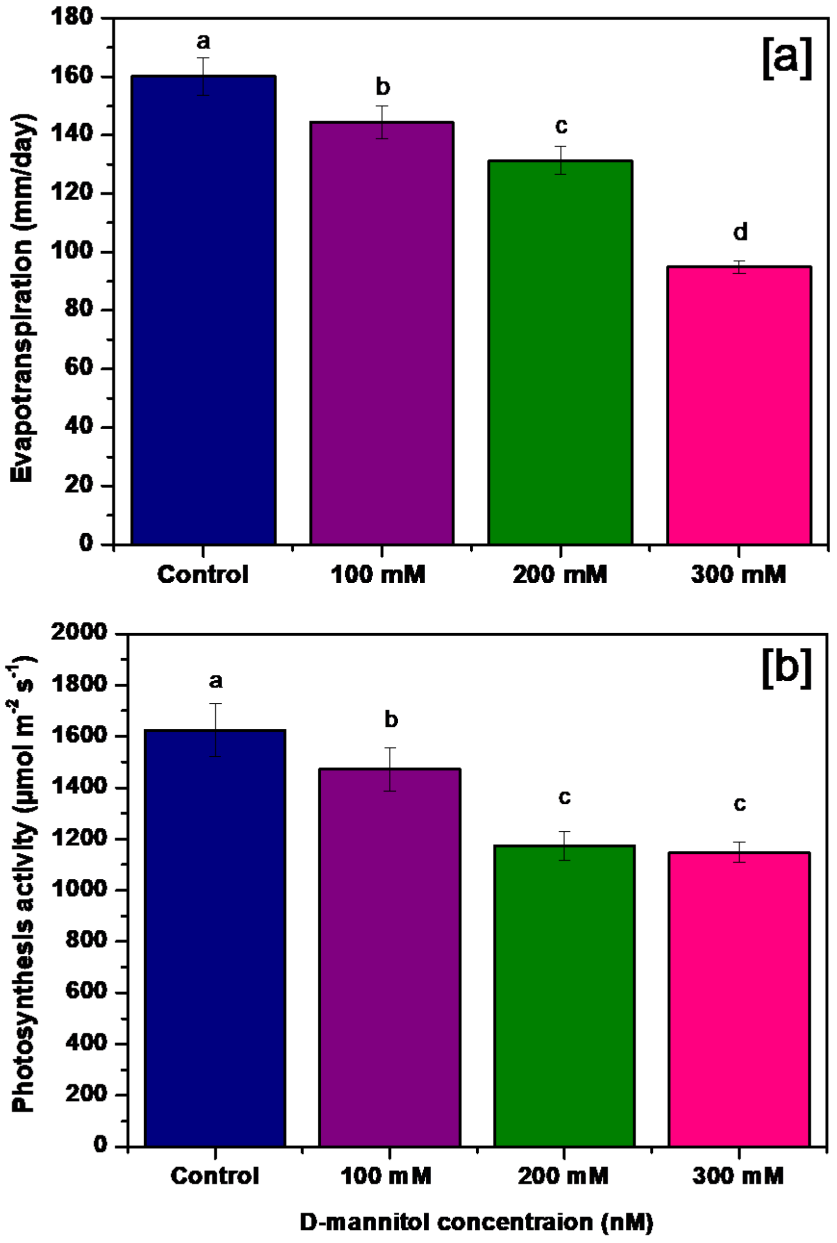

Figure S2. Mean effect of the drought stress level (0, 100, 200, 300 mM of D-mannitol) on real evapotranspiration (**A**) and photosynthetic activity (**B**), measured at 45 days after transplantation. Different letters above the vertical bars indicate significant difference at $p < 0.05$, according to Duncan's Multiple Range test.
